# Advances in understanding the mitochondrial mechanisms underlying acupuncture therapy for post-stroke cognitive impairment

**DOI:** 10.3389/fneur.2026.1806183

**Published:** 2026-05-08

**Authors:** Xiao Luo, Yuting Dong, Ziqi Chen, Yijing Jiang, Tao Chen, Liu Wu, Cheng Xie, Qinjian Dong, Xin Mu, Juan Li, Rongjiang Jin

**Affiliations:** 1 Department of Neurology, Chengdu Integrated TCM & Western Medicine Hospital, Chengdu, China; 2 School of Acupuncture and Tuina, Chengdu University of Traditional Chinese Medicine, Chengdu, China; 3 Department of Radiology, West China Hospital of Sichuan University, Chengdu, China; 4 Affiliated Rehabilitation Hospital of Fujian University of Traditional Chinese Medicine, Fuzhou, China; 5 School of Health Preservation and Rehabilitation, Chengdu University of Traditional Chinese Medicine, Chengdu, China; 6 Affiliated Rehabilitation Hospital of Chengdu University of Traditional Chinese Medicine/Sichuan Provincial BAYI Rehabilitation Center, Chengdu, China

**Keywords:** acupuncture, cognitive impairment, mitochondria, post-stroke cognitive impairment, stroke

## Abstract

Post-stroke cognitive impairment (PSCI) is a prevalent complication among stroke survivors. The core pathology of PSCI involves mitochondrial dysfunction. This review proposes that acupuncture may act as a multi-target regulator of mitochondrial homeostasis, potentially ameliorating PSCI through mechanisms such as improving core mitochondrial functions, including alleviating oxidative stress, correcting energy metabolism disorders, reducing calcium overload, stabilizing mitochondrial membrane potential (MMP), and repairing mitochondrial ultrastructure, regulating the mitochondrial quality control (MQC) system, mainly manifested in the bidirectional regulation of mitophagy, and inhibiting downstream pathological responses (neuroinflammation and apoptosis). However, high-quality clinical evidence establishing a causal link directly demonstrating that acupuncture improves PSCI by modulating mitochondrial function is extremely scarce. Clinical translation faces challenges including a lack of assessment tools and significant heterogeneity in treatment protocols. Future rigorously designed human studies are urgently needed to validate this mechanistic pathway and explore its translational potential in protocol optimization and combination therapies in PSCI patients.

## 1 Introduction

Post-Stroke Cognitive Impairment (PSCI) refers to cognitive decline occurring within the first six months following a stroke and is one of the major complications of stroke (1). According to the “Brief report on stroke prevention and treatment in China, 2024”, the global burden of disease study (GBD) of 2021 showed there were 2.77 million new cases of ischemic stroke in China, with a prevalence rate of 20.8 million cases and 1.18 million deaths caused by ischemic stroke (2). Approximately one-third of stroke patients experience PSCI, which significantly decreases patients’ self-care ability, quality of life and survival time (3). Consequently, the development of effective interventions for PSCI has emerged as a critical priority in both clinical practice and research.

Mitochondria serve as the central hub for cellular respiration and energy production in eukaryotic cells (4). Mitochondrial dysfunction emerges as the primary instigator in the post-stroke pathological cascade, playing a central role in ischemic–hypoxic injury through multiple interconnected mechanisms. Specifically, it participates in multiple pathological processes, such as structural alterations, disrupted energy metabolism, oxidative stress, calcium overload, mitochondrial membrane potential (MMP) changes, neuroinflammation, mitochondrial quality control (MQC) disorders and apoptosis. These maladaptive processes collectively contribute to the development and progression of PSCI (5, 6). Therefore, restoring mitochondrial homeostasis represents a critical therapeutic strategy for PSCI. Growing evidence positions mitochondria as a pivotal intervention target for PSCI (7–9).

Acupuncture, a classical therapy in traditional Chinese medicine, has demonstrated significant efficacy in ameliorating cognitive function in PSCI. Accumulating preclinical evidence indicates that acupuncture exerts therapeutic effects in PSCI through mitochondrial pathway modulation, including restoring ultrastructure changes, energy metabolism and MMP changes, modulating oxidative stress, neuroinflammation and apoptosis, and improving MQC dysfunction (10, 11). These synergistic mechanisms confer neuroprotection and enhance cognitive function through the amelioration of mitochondrial dysfunction (12). Previous studies have confirmed that acupuncture is beneficial for vascular dementia (VD) (13), Alzheimer’s disease (AD) (14), Parkinson’s disease (PD) (15), peripheral neuropathy (16), and neuropathic pain (17) through mitochondrial modulation. Mitochondria have been increasingly recognized as a critical therapeutic target in neurological disorders, with growing evidence suggesting that acupuncture mediates its neuroprotective effects through the modulation of mitochondrial function. Despite growing interest in the use of acupuncture for PSCI, the specific role of mitochondrial mechanisms in mediating the cognitive benefits of acupuncture remains unclear. This review aims to systematically examine the mechanisms of acupuncture treatment on mitochondrial dysfunction in PSCI, and summarize the effect of acupuncture on mitochondrial mechanisms such as mitochondrial structure changes, energy metabolism disorder, oxidative stress, calcium overload, MMP changes, neuroinflammation, defective MQC, and apoptosis in PSCI.

## 2 Acupuncture improves mitochondrial Core dysfunction in PSCI

### 2.1 Acupuncture restoration of mitochondrial structural alterations of in PSCI

Mitochondrial ultrastructural integrity is fundamental for sustaining neuronal bioenergetics and cellular homeostasis. In experimental models of PSCI, transmission electron microscopy analyses consistently demonstrate three hallmark ultrastructural pathologies: swollen mitochondria, disrupted cristae, and vacuolization (18–21). These morphological alterations serve as pathognomonic indicators of bioenergetic dysfunction and represent early features of neurodegenerative processes. Experimental studies have revealed that characteristic mitochondrial ultrastructural abnormalities, including mitochondrial swelling, ridge disappearance, and disordered arrangement, appeared in hippocampal neurons of male Wistar rats subjected to bilateral common carotid artery occlusion (BCCAO) (22). Notably, Tang et al. (23) found that the preservation of mitochondrial ultrastructural integrity in hippocampal neurons of the 2-vessel occlusion (2VO) rat model of vascular dementia correlated with significant cognitive recovery, as demonstrated by improved performance in the Morris Water Maze (MWM) navigation task.

Mounting evidence suggests that acupuncture may ameliorate cognitive function of PSCI by restoring mitochondrial ultrastructural integrity. Jia et al. (19) reported that electroacupuncture (EA) enhanced spatial memory and learning ability by MWM test in BCCAO rats by modulating mitochondrial energy metabolism, while also mitigating ultrastructural damage such as mitochondrial swelling, disruption of inner and outer membranes, and degeneration of cristae. Zhang et al. (24) found that EA improved cognitive ability of middle cerebral artery occlusion (MCAO) rats through inhibition of Ca^2+^ overload and restoration of mitochondrial ultrastructure integrity. These beneficial effects were reflected in decreased neurological deficit scores, shorter escape latency and reduced total distance traveled in the MWM, fewer electric shocks in the step-down test, and increased platform crossing times in the MWM. Zhong et al. (25) revealed that EA ameliorated cognitive impairment in MCAO rats, as evidenced by improved performance in the MWM test. This neuroprotective effect was mediated through the modulation of neuroinflammatory responses and mitigation of mitochondrial swelling, disorganized cristae, and partial cristolysis. Qiu et al. (26) showed that EA enhanced cognitive ability in BCCAO rats through attenuating neuroinflammation and ameliorating mitochondrial cristae disorganization. Lou et al. (27) observed that MCAO rats exhibited significant cognitive impairment in the MWM test, accompanied by pronounced mitochondrial structural alterations including swelling, cristae disruption, outer membrane fragmentation, matrix disorganization with vacuolization, and degranulation of rough endoplasmic reticulum, these pathological features were absent in sham-operated controls. Notably, acupuncture treatment attenuated these cognitive deficits through enhanced mitochondrial biogenesis, increased mitochondrial DNA (mtDNA) copy number, and preservation of ultrastructural integrity. Chen et al. (28) found that EA ameliorated learning and memory deficits in middle cerebral artery occlusion/reperfusion (MCAO/R) rats through modulation of mitochondrial dynamics, attenuating excessive fragmentation and maintaining ultrastructural integrity. Wang et al. (29) detected that EA improved cognitive behavior in MWM test and reduced neurologic deficit scores of MCAO rats, potentially through enhanced mitophagy and restoration of mitochondrial ultrastructure. Collectively, these findings demonstrate that acupuncture exerts multimodal regulation of mitochondrial structural stability in PSCI through preservation of cristae integrity, restoration of matrix organization and maintenance of outer membrane continuity. These ultrastructural improvements are positively correlated with enhanced synaptic plasticity and cognitive recovery, suggesting that mitochondrial structural stabilization constitutes an underlying acupuncture therapeutic mechanism in PSCI treatment.

### 2.2 Acupuncture ameliorates mitochondrial bioenergetic dysfunction in PSCI

Mitochondrial oxidative phosphorylation (OXPHOS) serves as the primary mechanism for ATP production in neurons, supplying the vital energy necessary for essential neurophysiological functions such as synaptic transmission, the maintenance of plasticity, and the propagation of action potentials (30, 31). In PSCI, ischemia-induced OXPHOS dysfunction triggers bioenergetic failure, culminating in ATP depletion. This energy deficit precipitates the collapse of ionic gradients and membrane depolarization, which unleashes a cascade of pathogenic events, including excitotoxicity, intracellular acidosis, calcium overload, and oxidative stress, ultimately culminating in neuroinflammation (32). As the central bioenergetic hubs of eukaryotic cells, mitochondria sustains extensive, multi-focal damage during ischemia–reperfusion injury, compromising both their functional integrity and ultrastructural organization (32). In the brain, the mitochondrial respiratory chain complexes I (NADH dehydrogenase), II (succinate dehydrogenase), and IV (cytochrome c oxidase) are particularly vulnerable to ischemic injury. This susceptibility leads to a marked decline in their enzymatic activity, which in turn impairs oxidative phosphorylation and exacerbates neuronal damage (33). In BCCAO rats, researchers observed a significant decline in spatial learning and memory abilities, accompanied by decreased activities of mitochondrial respiratory complex enzymes (I, II, and IV) and reduced protein expression of cyclooxygenase (COX) IV in hippocampal mitochondria (13).

Evidence indicates that acupuncture may improve cognitive function after stroke by restoring mitochondrial ATP production, thereby enhancing neuronal energy supply and ameliorating neurobehavioral deficits. Jia et al. (19) revealed that EA ameliorated cognitive dysfunction in BCCAO rats by restoring mitochondrial energy metabolism. Mechanistically, EA restored mitochondrial function by enhancing the activity of respiratory complexes I-IV, boosting ATP production, repairing ultrastructural damage, and preserving fatty acid oxidation capacity. Furthermore, hippocampal mitochondrial complex I levels were positively correlated with cognitive performance, underscoring their pivotal role in EA-mediated neuroprotection. In a rat model of multi-infarct dementia (induced by carotid artery microembolization), acupuncture treatment reversed mitochondrial dysfunction by enhancing respiratory chain enzyme activity, promoting oxidative phosphorylation efficiency, and ultimately resulting in increased ATP production (34). These improvements in mitochondrial bioenergetics were associated with significantly shortened escape latency in the MWM test, indicating enhanced spatial learning ability. Li et al. (13) found that EA ameliorated spatial learning and memory deficits in BCCAO rats, which was reflected by enhanced performance in the MWM test. This cognitive enhancement was mediated through upregulation of mitochondrial respiratory chain complex activities and increased expression of COX. Similarly, acupuncture treatment was found to enhance mitochondrial biogenesis and significantly elevate ATP levels in MCAO rats. These improvements showed a negative correlation with neurological deficit scores and cerebral infarction volume, while demonstrating a positive correlation with cognitive performance in the MWM test (27). EA treatment significantly ameliorated learning and memory deficits in MCAO/R rats by regulating mitochondrial dynamics. This beneficial effect was accompanied by enhanced ATP production and upregulated activity of COX I-IV in the mitochondrial respiratory chain (28). As mentioned above, acupuncture could lead to neuroprotection through coordinated regulation of neuronal mitochondrial energy metabolism. Specifically, it involves modulating key components of the oxidative phosphorylation system, including electron transport chain complexes, ATP synthase, and COX. This multi-targeted enhancement of mitochondrial bioenergetic capacity helps mitigate ischemic neuronal damage and ameliorates associated cognitive dysfunction.

### 2.3 Acupuncture mitigates mitochondrial oxidative stress in PSCI

Oxidative stress plays a pivotal role in the pathogenesis of PSCI, where mitochondria serves as both primary generators and key targets of reactive oxygen species (ROS)-mediated damage. As the predominant cellular source of ROS, mitochondria are particularly vulnerable to oxidative injury, initiating a detrimental cycle of mitochondrial dysfunction and neuronal degeneration that ultimately results in neuronal death and cognitive impairment (35, 36). Under ischemic conditions, mitochondrial bioenergetic dysfunction triggers excessive ROS production, overwhelming endogenous antioxidant defenses. This results in impaired clearance of oxygen free radicals and subsequent oxidative damage to cellular components, exacerbating ischemic injury (37). The gastrin-releasing peptide agonist demonstrated significant neuroprotective effects in MCAO rats, ameliorating cognitive deficits and synaptic plasticity impairments. These protective effects were mediated via modulation of oxidative stress, including the restoration of total superoxide dismutase (SOD) and catalase (38), and the reduction of malondialdehyde (MDA) levels (39). Therefore, therapeutic strategies that target mitochondrial protection and mitigate post-ischemic oxidative stress may represent a promising neuroprotective approach for preserving neuronal viability and promoting cognitive recovery.

Acupuncture’s dual capacity to mitigate oxidative injury and enhance endogenous antioxidant defenses makes it a promising therapeutic strategy for both neuroprotection and cognitive recovery in PSCI. Lin et al. (40) reported that EA significantly improved the neurological deficit score and increased MWM platform crossing times, while reducing MWM escape latency. Furthermore, EA elevated mitochondrial SOD and GSH-Px activities and lowered MDA levels in the cerebral cortex, thereby attenuating oxidative stress-induced damage. Jittiwat et al. (41) found that laser acupuncture at Baihui (GV 20) alleviated memory deficits in MCAO rats, as measured by improved performance in the MWM test. The underlying neuroprotective mechanism involved enhanced mitochondrial SOD activity, upregulated hippocampal GSH-Px function, and preserved neuronal density in the hippocampal CA1 and CA3 subregions. Du et al. (42) reported that EA significantly restored the activity of antioxidant enzymes, elevated SOD levels, and reduced ROS production along with the expression of thioredoxin-interacting protein, nucleotide-binding oligomerization domain-like receptor protein 3 (NLRP3), caspase-1 and IL-1β. These changes ultimately led to a decrease in cerebral infarction size and alleviated cognitive dysfunction in vascular dementia (VD) rats induced by 2-vessel occlusion (2VO). Li et al. (13) discovered that EA ameliorated cognitive deficits in BCCAO rats by modulating mitochondrial energy metabolism and reducing ROS production. Zhang et al. (34) reported that acupuncture shortened MWM escape latency of multi-infarct dementia (MID) rats by improving mitochondrial energy metabolism and attenuating oxidative stress. Su et al. (43) found that acupuncture inhibited mitochondrial membrane depolarization and oxidative stress in MCAO rats through reducing ROS levels and increasing SOD activity. These beneficial effects were associated with improved cognitive performance, as demonstrated by decreased escape latency and increased platform crossing frequency in the MWM test. Li et al. (44) proposed that acupuncture could improve the cognitive function of BCCAO rats by activating the Phosphatidylinositol 3-Kinases (PI3K)/protein kinase B (Akt)/mammalian target of rapamycin (mTOR) signaling pathway and attenuating oxidative stress via restoration of hippocampal redox homeostasis, characterized by reduced ROS and MDA levels alongside increased SOD activity. Zhong et al. (25) and Qiu et al. (26) showed that EA improved cognitive ability of BCCAO rats through attenuation of ROS production, suppression of inflammasome activation, and restoration of mitophagy processes. Luo et al. (45) also found that EA improved neurological deficit scores, reduced cerebral infarction volume, and enhanced cognitive performance in the MWM test of MCAO rats. These therapeutic benefits were mediated through modulation of the inflammation-autophagy axis and correction of oxidative imbalance, as evidenced by decreased MDA levels and restored SOD activity. Lou et al. (27) found that the neurorestorative effects of acupuncture in MCAO rats involved enhanced mitochondrial biogenesis and redox regulation, marked by upregulation of SOD/GSH and reduction of MDA, ultimately improving neurological and cognitive function. Chen et al. (28) further revealed that EA mitigated oxidative stress in MCAO/R rats by reducing MDA levels and elevating SOD activity. These changes were correlated with enhanced cognitive performance, as indicated by higher discrimination indices (DI) in the novel object recognition (NOR) test, and were also associated with the regulation of mitochondrial dynamics. Lin et al. (46) reported that EA improved neurologic deficit scores, as well as learning and memory behaviors in MWM test. These therapeutic effects were mediated through the attenuation of oxidative stress, restoration of calcium pump homeostasis, and suppression of apoptotic factor release.

In summary, acupuncture contributes to restoring mitochondrial function by enhancing antioxidants (SOD, GSHPx, GSH/GSSG), reducing oxidants (ROS, MDA), and preventing mitochondrial dysfunction, neuroinflammation, and apoptosis. However, current evidence demonstrates that acupuncture ameliorates oxidative stress through multiple signaling pathways and molecular targets (47), however, the essential role of mitochondrial oxidative stress pathways in acupuncture’s therapeutic effects on PSCI requires further investigation.

### 2.4 Acupuncture attenuates calcium overload in PSCI

Under physiological conditions, the uptake and excretion of mitochondrial calcium are maintained through the continuous and slow circulation of Ca^2+^ through the inner membrane of mitochondria. Mitochondrial Ca^2+^ not only augments oxidative phosphorylation to drive ATP production but also modulates the electrochemical potential across the inner mitochondrial membrane, thereby ensuring efficient energy metabolism (48). Elevated calcium levels trigger the opening of the mitochondrial permeability transition pore (MPTP) in the inner mitochondrial membrane, allowing solutes to enter the matrix. This influx creates an osmotic imbalance, which causes matrix swelling, rupture of the mitochondrial membranes, and activation of the intrinsic apoptosis pathway (49). Concurrently, this process initiates downstream ischemic cascades, which ultimately disrupt intracellular signaling pathways and compromise cellular structural and functional integrity (32). Moreover, elevated intracellular Ca^2+^ may suppress mitochondrial respiration (50), induce endoplasmic reticulum associated mitochondrial membrane dysregulation, leading to mitochondrial dysfunction (51) and subsequent neurodegeneration (52).

Acupuncture confers neuroprotective and cognitive-preserving effects by mitigating calcium overload and modulating downstream cellular responses. Within this process, the calmodulin (CaM)-calmodulin dependent protein kinase (CaMK) Type IV -cyclic adenosine monophosphate response element-binding protein (CREB) signaling pathway plays a key role in mitochondrial homeostasis (53) and supports learning and memory function (54). Zhang et al. (24) found that EA lowered the MWM escape latency, the total distance and the number of electric shocks in the step-down test in MCAO rats. Additionally, EA increased the number of platform crossings in the MWM and decreased Ca^2+^ levels in the hippocampal CA1 region. Han et al. (55) revealed that EA improved the neurological deficit score and shortened escape latency of MCAO rats in MWM, potentially through promoting synaptogenesis in the CA1 region and modulating the CaM-CaMKII-CREB signaling pathway. Zhang et al. (56) reported that EA effectively inhibited the expression and activity of CaM, enhanced the expression and phosphorylation of CaMKIV and CREB, thus improving the step-down latency of step-down avoidance test, decreased the error numbers and infarction volume of MCAO rats. Dai et al. (57) revealed that EA increased the protein expression and phosphorylation levels of NMDAR, *α*-amino-3-hydroxy-5-methyl-4-isoxazolpropionate receptor and CaMK II, and improved the learning and memory behaviors in MWM test of VCI rats (2VO). Taken together, the evidence suggests that acupuncture participates in mitigating PSCI primarily through modulation of the CaM/CaMK/CREB signaling pathway. This action could exert neuroprotective effects by suppressing mitochondrial apoptosis and Ca^2+^-dependent neurotoxicity, while concurrently enhancing synaptic plasticity and promoting synaptic reconstruction in cognition-related networks.

### 2.5 Acupuncture maintains membrane potential stability in PSCI

Maintaining MMP is crucial for preserving ionic/metabolic balance and overall mitochondrial function. Its disruption, primarily triggered by the opening of MPTP, leads to irreversible damage through membrane depolarization and the release of pro-apoptotic factors (58). The depolarization of MMP and the activation of MPTP are key hubs of apoptosis and inflammation, which is closely related to the integrity of mitochondrial ultrastructure and function (59). Increased mitochondrial outer membrane permeabilization (MOMP) results in the release of pro-apoptotic proteins from the mitochondrial intermembrane space, ultimately triggering apoptotic cell death and contributing to the onset of various neurodegenerative diseases (60). MPTP dysfunction has been implicated in synaptic and cognitive impairment associated with ischemia/reperfusion injury (61, 62).

By targeting MMP depolarization and MPTP opening, acupuncture inhibits key drivers of neuronal damage in PSCI, thereby preventing apoptosis and improving cognitive outcomes. By inhibiting the function of translocase of outer mitochondrial membrane 40 and inner mitochondrial membrane 17A, acupuncture improved MWM performance in MCAO rats, as evidenced by shorter escape latency and more frequent platform crossings (43). Li et al. (44) proposed that acupuncture shortened MWM escape latency of VD rats (BCCAO) by activating the PI3K/Akt/mTOR signaling pathway. This activation leads to the inhibition of MPTP opening and the alleviation of oxidative stress in the hippocampus, collectively contributing to cognitive improvement. Li et al. (13) demonstrated that EA treatment not only significantly shortened MWM escape latency in BCCAO rats but also restored mitochondrial energy metabolism and stabilized MMP. Lou et al. (27) revealed that acupuncture ameliorated neurological deficits, reduced infarct size, and improved learning and memory in MCAO rats via a mitochondrial protective mechanism involving enhanced biogenesis, increased copy number, and suppressed MMP depolarization. Collectively, acupuncture offers neuroprotection after stroke by stabilizing MMP, restoring mitochondrial function, and reducing apoptosis, thus improving cognitive recovery.

## 3 Acupuncture modulates mitochondrial quality control system in PSCI

The MQC system, encompassing biogenesis, dynamics (fusion/fission) and mitophagy, is a crucial endogenous mechanism for maintaining mitochondrial homeostasis (63). By regulating these essential cellular processes, MQC has been showed to attenuate cerebral ischemic injury and promote post-stroke neurological and cognitive recovery (5, 64, 65).

### 3.1 Acupuncture promotes mitochondrial biogenesis and dynamics in PSCI

Mitochondrial biogenesis refers to the growth and division of mitochondria, encompassing the production of the inner and outer membranes of the mitochondria, the replication of the genome, and the synthesis and assembly of mitochondrial proteins (66). PGC-1α is a central transcriptional regulator in mitochondrial biogenesis, driving the process by activating protein transcription and mitochondrial DNA (mtDNA) replication (63). A study has indicated that the improvement of cognitive dysfunction via enhanced mitochondrial biogenesis in transient middle cerebral artery occlusion (tMCAO) rats was accompanied by the upregulation of PGC-1α and mitochondrial transcription factor A (TFAM), increased mtDNA content, and elevated ATP production (65). Mitochondrial dynamics sustains mitochondrial health through the continuous regulation of fusion and fission. This process enables the removal of damaged organelles, thereby preventing their harmful accumulation (67). Mitochondrial fusion is primarily regulated by Mitofusin (68) and Optic atrophy 1 (OPA1) (69), while mitochondrial fission is governed by key factors including Dynamin-related protein 1 (DRP1) (70) and factors associated with the endoplasmic reticulum and S-OPA1 (a fragmented form of OPA1) (63).

By promoting mitochondrial biogenesis, acupuncture has been reported to enhance cerebral tolerance to ischemia–reperfusion injury (71). Lou et al. (27) found that acupuncture promoted mitochondrial biogenesis and improved neurobehavioral assessment scores and MWM performance in MCAO rats, significantly regulating the PGC-1α/NRF-1/TFAM pathway, which is essential for mitochondrial genesis and synthesis. Furthermore, acupuncture’s stabilization of mitochondrial dynamics was found to contribute to the restoration of neurological and cognitive function (71). By upregulating Sirt1, PGC-1α, and OPA1 and downregulating DRP1, EA regulated mitochondrial dynamics in MCAO/R rats, alleviating mitochondrial damage and fragmentation. These changes contributed to improved neurological function and cognitive performance, as indicated by reduced deficit scores and NOR DI (28).

### 3.2 The bidirectional regulatory role of acupuncture on mitophagy in PSCI

Ischemic injury induces mitochondrial damage and depolarization, which initiates selective macroautophagy. Damaged mitochondria are subsequetly encapsulated within autophagosomes, leading to lysosomal fusion and ultimately their degradation (72). Evidence showed that modulating mitophagy and mitochondrial apoptosis facilitated the recovery of neurological deficits and cognitive dysfunction in mice subjected to temporary bilateral common carotid artery occlusion (tBCCAO) (73).

Acupuncture functions as a bidirectional modulator of mitophagy to maintain mitochondrial homeostasis, thereby reducing neural damage. Wang et al. (29) discovered that EA reduced infarct volume and improved spatial learning in MCAO rats, as evidenced by shorter MWM escape latency and an increased number of platform crossings. This neuroprotection was attributed to the activation of the PTEN-induced kinase 1 (PINK1)/Parkin-mediated mitophagy pathway and the PI3K/AKT/mTOR/Beclin-1 signaling pathway, which enhanced mitophagic clearance of damaged mitochondria. Su et al. (74) reported that EA attenuated neurological deficits and restored learning and memory in MCAO/R rats via mitophagy upregulation, which involved elevated levels of PINK1, Parkin, Beclin-1, Sirt1, FOXO3, and the LC3 II/I ratio, coupled with suppressed P62 activation. Li et al. (44) proposed that acupuncture enhanced spatial memory in BCCAO rats as assessed by the MWM test, potentially through the mitigation of oxidative stress and activation of the hippocampal PI3K/Akt/mTOR signaling pathway. Zhong et al. (25) found that EA reduced cerebral infarction area and improved spatial learning performance in MCAO rats, as evidenced by shorter escape latency and reduced total distance traveled in the MWM test. These benefits were attributed to the upregulation of mitophagy-associated proteins, including PINK1, Parkin, Beclin-1 and the LC3 II/LC3 I ratio, along with the suppression of inflammatory responses.

On the other hand, it has been reported that EA improve spatial learning and memory in BCCAO rats, as reflected by shortened escape latency and increased platform crossings in the MWM test. These improvements are achieved through the inhibition of inflammatory responses and autophagy, accompanied by reductions in the number of autophagosomes, the LC3-II/LC3-I ratio, and Beclin1 protein expression (26). Lang et al. (75) found that EA down-regulated the LC3 II/LC3 I ratio, up-regulated the expression of p62, mTOR and Beclin-1, and reduced the number of autophagosomes in MCAO rats, thereby ameliorating neurologic deficits and reducing the infarction area, while also reducing escape latency and total distance, increasing MWM platform crossing time and prolonging time in the target quadrant during the MWM test. Luo et al. (45) also reported that EA improved the neurologic deficit and MWM test scores of MCAO rats by regulating the mTOR/NLRP3 pathway, thus inhibiting autophagy and neuroinflammation.

To sum up, acupuncture suggests a role for improving neurological function and cognitive deficits in PSCI by modulating mitochondrial biogenesis and dynamics. However, current evidence remains limited and insufficiently robust to support a definitive conclusion. As is well established, mitophagy serves as a homeostatic mechanism that eliminates damaged mitochondria to maintain cellular health, yet excessive activation may result in cellular impairment. Acupuncture appears to exert a bidirectional regulatory influence on mitophagy homeostasis, which could underlie its therapeutic potential. Nevertheless, its specific impact on cognitive function in PSCI warrants further investigation.

## 4 Acupuncture mitigates mitochondrial dysfunction-induced pathological cascades in PSCI

### 4.1 Acupuncture attenuates mitochondria-driven neuroinflammation in PSCI

Mitochondria plays an important role in the initiation of inflammation (76). Certain mitochondrial components, such as those acting as pattern recognition receptor (PRR) (77), damage-associated molecular patterns (mtDAMPs) can directly activate their corresponding PRRs and induce inflammatory response (78). Additionally, MOMP during regulated cell death triggers autophagy and caspase activation, further contributing to the inflammatory cascade (79, 80). Evidence indicates that elevated levels of serum mitochondrial DNA are closely associated with post-ischemic neuroinflammation (81, 82). In addition, studies have shown that mitochondrial reactive oxygen species (mtROS) and mtDNA can activate the NLRP3 inflammasome (83, 84), thereby promoting neuroinflammation (85). Notably, this process could be attenuated through the enhancement of mitophagy (86, 87). A recent study suggested that while both neuroinflammation and mitochondrial dysfunction were independently associated with cognitive decline, they might not be directly causally related to each other (88). Zhao et al. (89) found that cognitive dysfunction in a chronic cerebral hypoperfusion (CCH) model (BCCAO rats) was ameliorated by activation of mitochondrial protection and suppression of neuroinflammation. Therefore, neuroinflammation significantly contributes to the pathological progression of PSCI. However, mitochondrial dysfunction and neuroinflammation appear to mutually reinforce and exacerbate one another, potentially leading synergistically to neurocognitive impairment (90). Nevertheless, mitochondrial dysfunction is likely not the sole driver of neuroinflammation.

Zhong et al. (25) found that EA decreased infarction area, MWM escape latency and total distance of MCAO rats by up-regulating mitophagy associated proteins and inhibiting ROS-induced NLRP3 inflammasome activation. Qiu et al. (26) showed that EA alleviated mitochondrial ridge structure disorder, reduced autophagosome number and the expression of ROS, NLRP3, Beclin1 and LC3-II/LC3-I ratio, eventually improving escape latency and platform crossing times assessment in MWM test of BCCAO rats. Lang et al. (75) found that EA down-regulated the expression of IL-1β, IL-18, NLRP3 and LC3 II/LC3 I ratio in MCAO rats, while up-regulated the expression of p62, mTOR and Beclin-1. This modulation of the autophagy-inflammation pathway contributed to improving neurological function and enhancing cognitive performance in the MWM test. Similarly, Luo et al. (45) also found that EA improved the neurologic deficit scores, MWM escape latency, and platform crossing times of MCAO rats by regulating the mTOR/NLRP3 autophagy-inflammation pathway. Jittiwat et al. (41) and Du et al. (42) reported that acupuncture improved cognitive function in PSCI rat models (MCAO and 2VO) by suppressing oxidative stress and inflammation, along with reducing levels of IL-6, IL-18 and ROS.

In summary, acupuncture is beneficial to enhance mitochondrial function and modulate neuroinflammatory pathways, thereby reducing the release of downstream inflammatory factors in PSCI. Acupuncture is also considered to alleviate neuroinflammation through immune (91, 92), cholinergic anti-inflammatory (93), and other multiple pathways (12), ultimately ameliorating neurological damage and cognitive impairment in PSCI. Although neuroinflammation and mitochondrial dysfunction may mutually promote each other (90), whether the anti-neuroinflammatory effect of acupuncture directly depends on mitochondrial regulation requires further validation.

### 4.2 Acupuncture regulates mitochondrial apoptotic pathways in PSCI

Mitochondria act as a regulatory center of apoptosis and can mediate multiple apoptotic pathways. Following ischemic stimulation, mitochondria release various pro-apoptotic factors. These factors activate the intracellular apoptotic protease Caspase, which in turn acts upon mitochondria, leading to further release of pro-apoptotic factors and ultimately resulting in cellular apoptosis and necrosis (94). The opening of MPTP, a key factor leading to apoptosis, is inhibited by anti-apoptotic protein Bcl-2 and promoted by the pro-apoptotic protein Bax (95). Studies have found that suppressing mitochondrial apoptosis and modulating the expression of caspase-3, Bax and Bcl-2 can significantly improve neurobehavioral and cognitive function in MCAO rats (96, 97).

Acupuncture has been shown to improve neurological and cognitive outcomes in PSCI by regulating apoptotic pathways. However, whether these pathways are linked to mitochondrial dysfunction remains a pertinent question. It has been reported that EA improved learning and memory capacity, as assessed by the MWM test, also reduced cerebral infarction volume and neurological deficit scores in MCAO rats. These effects are achieved through the inhibition of mitochondrial apoptosis, coupled with significant activation of the cAMP/CREB signaling pathway and an increased Bcl-2/Bax ratio (46). Research indicated that EA alleviated apoptosis and neuron loss, and also improved neuromotor function and Rota-rod test performance in MCAO rats. These beneficial effects were mediated through the inhibition of Cofilin formation and mitochondrial translocation, as well as the suppression of Caspase-3 cleavage (98). Lin et al. (40) showed that EA reduced the neurological deficit score and shortened MWM escape latency of MCAO rats by alleviating apoptosis and oxidative stress, along with reducing caspase-3 and MDA levels in cortical mitochondria. Evidence has shown that acupuncture regulates apoptosis during neurocognitive recovery, although a direct mitochondrial-related mechanism has not yet been clearly established. Shi et al. (99) revealed that EA inhibited apoptosis, up-regulated Adenosine Receptor 1 and the ratio of Bcl-2/Bax in MCAO rats. These changes were associated with improved neurological and cognitive function, as reflected by reduced deficit scores, shorter escape latency, higher mean speed, and longer time spent in the target quadrant of the MWM test. Yun et al. (100) discovered that laser acupuncture activated CRE-mediated apoptosis pathways, upregulated Bcl-2, downregulated Bax, and shortened MWM escape latency of MCAO rats. Lang et al. (75) found that EA downregulated inflammatory and autophagy markers, improved neurobehavioral outcomes and cognitive performance in the MWM test, and reduced caspase-3 expression, Bax levels and apoptotic cell counts while upregulating Bcl-2 expression of MCAO rats.

As summarized above, acupuncture may contribute to the inhibition of the apoptotic cascade induced by the release of mitochondrial components, thereby balancing apoptotic factors and stabilizing the MPTP. Simultaneously, acupuncture modulates pathways involved in oxidative stress, inflammation and apoptosis. These regulatory effects may operate in parallel with its mitochondria-targeting mechanisms; however, the interrelationships require further exploration.

## 5 Summary

As in Table 1, the reviewed preclinical studies predominantly utilize rodent models of cerebral ischemia, such as BCCAO, pBCCAO and MCAO. The primary acupuncture modalities employed are manual acupuncture (MA) and EA, with specific parameters (e.g., frequency, intensity, duration) varying across studies. The research mechanisms focus extensively on mitochondrial function and related pathways. These mitochondrial interventions are consistently linked to improved outcomes in neurological and cognitive behavioral tests, such as the MWM and NOR test. The primary mitochondrial outcomes assessed include biomarkers of oxidative stress (e.g., SOD, MDA, GSH), apoptosis (e.g., Bcl-2/Bax, Cyto C), energy metabolism (e.g., ATP levels, OXPHOS complexes), and synaptic plasticity proteins.

**Table 1:** Summary of preclinical studies on acupuncture, mitochondria, and post-stroke cognitive impairment.

Num	Study	Model	Acupoints	Acupuncture modality/parameter	Main mechanism associated with mitochondria	Neurological and cognitive behavioral results	Primary mitochondrial outcomes assessed
1	Zhang et al. (34)	MID	CV17, CV12, CV5, SP10, ST36	MA, twisting 2 ~ 3 times/s, 30s/day, 21 days.	Mitochondrial respiratory dysfunction, oxidative stress and cognitive ability	MWM escape latency↓	CBF↑, total SOD↑, CuZnSOD↑, MnSOD↑, GSH↑, GSSG↓, MDA↓, O2-↓, RCI↑, P/O ratio↑, mitochondrial respiratory complex I and IV activities↑
2	Lin et al. (40)	MCAO	GV24, GV20	EA, frequency 5 ~ 20 Hz, intensity 3 mA, 30 min/day, 7 days.	Oxidative stress, apoptosis and cognitive ability	Neurologic deficit scores↓, infarction volume↓, MWM escape latency↓, MWM total distance↓, MWM platform crossing times↑	MDA↓, SOD↑, GSHPx↑, p-CREB↑, Bcl-2↑, Bax↓
3	Zhang (24)	MCAO	GV24, GV20	EA, dense-sparse wave, frequency 2 ~ 20 Hz, intensity 2 mA, 30 min/day, 7 days.	Calcium overload and cognitive ability	Neurological deficit score↓, MWM escape latency↓, MWM total distance↓, MWM platform crossing times↑, the step-down test: electric shock number↓, infarction volume↓	Nissl body positive cells number↑, the integrity of the envelope, chromatin and mitochondrial ultrastructure↑, Glu↓, the protein expression of NR1 and NR2B↓, the protein expression of NR2A↑, Ca2 + ↓
4	Lin et al. (40)	MCAO	GV24, GV20	EA, dense-sparse wave, frequency 1 ~ 20 Hz, voltage 3 ~ 5 V, 30 min/day, 7 days.	Oxidative stress, apoptosis and cognitive ability	Neurological deficit score↓, MWM escape latency↓, MWM platform crossing times↑	caspase-3↓, MDA↓, SOD↑, GSH-PX↑
5	Li et al. (13)	BCCAO	GV20, ST36	MA, twisting 2 times/s, 30 s/day, 6 days/week, 2 weeks.	Mitochondrial respiratory dysfunction and cognitive ability	MWM escape latency↓	ROS↓, mitochondrial respiratory complex I, II and IV activities↑, mitochondrial RCR↑, MMP↑, COX IV↑
6	Zhang et al. (56)	MCAO	GV24, GV20	EA, disperse wave, frequency 1 ~ 20 Hz, 30 min/day, 7 days.	CaM-CaMKIV-CREB axis and cognitive ability	The step-down avoidance test: the step-down latency↑, error numbers↓, infarction volume↓	CaM↓, CaMKIV↑, p-CaMKIV↑, CREB↑, p-CREB↑
7	Yun et al. (100)	MCAO	GV20, HT7	LA, laser-guided needles, wavelength 650 nm, intensity 30 mW, frequency 100 Hz, 125 μm laser spot diameter, 5 min/day, once every other day, 2 weeks.	CREB and CRE-mediated pathways, apoptosis and cognitive ability	MWM escape latency↓	ChAT density↑, CREB↑, BDNF↑, Bcl-2↑, Bax↓
8	Du et al. (42)	2VO	GV20, ST36	MA, rotating 1 min, 1 time/day, 13 days/2 weeks.	Oxidative stress, inflammatory and cognitive ability	MWM escape latency↓, MWM total distance↓, MWM time spent↓	neuronal density in CA1 of the hippocampus↑, ROS↓, 8-OHdG↓, SOD↑, TXNIP↓, NLRP3↓, caspase-1↓, and IL-1β↓
9	Su et al. (43)	MCAO	GV20, GV14, GV16, GV26	MA, rotating, 20 min/day, 15 days.	Oxidative stress, mitochondrial membrane potential and cognitive ability	MWM escape latency↓, MWM platform crossing times↑	the integrity of cell structure↑, cell apoptosis number↓, ATP↑, SOD↑, NO↓, iNOS↓, mitochondrial membrane depolarization↓, ROS↓, TOMM40↓, TIMM17A↓, Aβ↓, APP↓, COX↑
10	Jittiwat (41)	MCAO	GV20	LA, pulsed waves, 100 mV, wavelength 810 nm, 100 μm laser spot diameter, 10 min/day, 14 days.	Oxidative stress, inflammatory and cognitive ability	Neurological deficit score↓, MWM escape latency↓, MWM retention time↑	neuronal density in CA1 and CA3↑, SOD↑, GSH-PX↑, IL-6↓
11	Wang et al. (3)	MCAO	GV24, GV20	EA, expanding wave, voltage 6 V, frequency 1 ~ 20 Hz, intensity 2 mA, 30 min/day, 8 days.	Mitophagy and cognitive ability	Neurologic deficit scores↓, infarction volume↓, MWM escape latency↓, MWM platform crossing times↑	the integrity of cell structure and mitochondrial ultrastructure↑, autophagosomes↑, PI3K↑, mTOR↑, Beclin-1↑, p-Akt↑, p53↓
12	Shi et al. (99)	MCAO	GV20	EA, disperse wave, frequency 2 ~ 15 Hz, intensity 1 mA, 90 min, 1 time.	Apoptosis and cognitive ability	MWM escape latency↓, MWM mean speed↑, MWM time in target quadrant↑, neurologic deficit scores↓	A1R protein expression↑, Bcl-2/Bax↑, CA1 neuron number↑
13	Chen et al. (98)	MCAO	GV24, GV20	EA, respectively 5 min and 6 h after the onset of MCAO, dense-sparse wave, frequency 4 ~ 20 Hz, intensity 1 mA, for 30 min.	Apoptosis and cognitive ability	Rota-rod test time↑, neurologic deficit scores↓, infarction volume↓	apoptotic cells number↓, caspase-3↓, cofilin rod↓, cofilin in mitochondria and cytoplasm↓
14	Li et al. (44)	pBCCAO	GV20, ST36	MA, 1 time/day, 13 days/2 weeks.	Oxidative stress, mitochondrial membrane potential, PI3 K/Akt/mTOR signaling pathway, and cognitive ability	MWM escape latency↓, MWM spatial memory ability↑	ROS↓, MDA↓, AChE↓, SOD↑, ChAT↑, MMP↑, p-PI3 K↑, p-Akt↑, mTOR↑
15	Zhong et al. (25)	MCAO	GV24, GV20	EA, dense-sparse wave, voltage 2 V, frequency 4 ~ 20 Hz, intensity 0.5 mA, 30 min/day, 7 days.	Inflammation, mitophagy and cognitive ability	Infarction volume↓, MWM escape latency↓, MWM total distance↓, MWM platform crossing times↑	AANAT↑, LC3-II/I↑, Beclin-1↑, PINK1↑, Parkin↑, the integrity of mitochondrial ultrastructure↑, ROS↓, NLRP3↓, ASC↓, IL-1β↓, IL-18↓, caspase-1↓, Lba-1↓
16	Qiu et al. (26)	BCCAO	GV14, GV20, BL23	EA, dense-sparse wave, frequency 10 ~ 50 Hz, intensity 1 mA, 30 min/day, 4 weeks.	Inflammation, mitophagy and cognitive ability	MWM escape latency↓, MWM platform crossing times↑	the integrity of mitochondrial ultrastructure↑, autophagosome↓, ROS↓, NLRP3↓, LC3-II/I↓, Beclin-1↓
17	Dai et al. (57)	2VO	GV24, GV20	EA, dense-sparse wave, voltage 6 V, frequency 2 ~ 20 Hz, intensity 1 ~ 3 mA, 30 min/day, 5 times/week, 4 weeks.	Glu, NMDAR and CAMKII signalling pathway, synaptic plasticity and cognitive ability	MWM escape latency↓, MWM platform crossing times↑	LTP↑, the frequency and amplitude of sEPSCs↑, the decay and rise times of sEPSCs↑, phosphorylation of NMDAR2B, GluR1, and CaMKII↑
18	Lou et al. (27)	MCAO	GV24, GV20	MA, twirling ±180°, frequency 1 Hz, stimulate 2 min followed by a 3 min-break, repeat 6 times during 30 min.	Mitochondrial biogenesis, oxidative stress and cognitive ability	Neurologic deficit scores↓, MWM escape latency↓, MWM platform crossing times↑, infarction volume↓	the integrity of cell structure and mitochondrial ultrastructure↑, PGC-1α↑, NRF-1↑, TFAM↑, mitochondrial OXPHOS↑, CI-NDUFB8↑, CIV-MTCO1↑, CV-ATP5A↑, mitochondrial copy number↑, MMP change↓, ATP↑, MAP2↑, MDA↓, SOD↑, GSH/GSSG↑
19	Chen et al. (28)	MCAO/R	GV24, GV20	EA, continuous wave, voltage 6 V, frequency 1 ~ 20 Hz, 30 min/day, 14 days.	Mitochondrial dynamics and cognitive ability	Neurologic deficit scores↓, NOR DI↑, infarction volume↓	the integrity of cell structure and mitochondrial ultrastructure↑, the number of mitochondria↓, SOD↑, ATP↑, enzyme activity of mitochondrial complex I ∼ IV↑, MDA↓, SIRT1↑, PGC-1α↑, OPA1↑, DRP1↓
20	Luo et al. (45)	MCAO	GV14, GV16, GB20	EA, expanding wave, voltage 6 V, frequency 1 ~ 20 Hz, intensity 2 mA, 30 min/day, 8 days.	Inflammation, autophagy and cognitive ability	Neurologic deficit scores↓, MWM escape latency↓, MWM platform crossing times↑, infarction volume↓	the integrity of hippocampus cell structure↑, TNF-α↓, IL-6↓, MDA↓, IL-10↑, SOD↑, LC3-II/I↓, caspase-3↓, Beclin1↓, Bax↓, NLRP3↓, Bcl-2↑, p62↑, mTOR↑
21	Lang et al. (75)	MCAO	GV14, GV16, GB20	EA, continuous wave, frequency 20 Hz, intensity 1 mA, 30 min/day, 14 days.	Inflammation, autophagy and cognitive ability	Body weight↑, neurologic deficit scores↓, MWM escape latency↓, MWM total distance↓, MWM platform crossing times↑, MWM time in target quadrant↑, infarction volume↓	apoptotic cells number↓, Bax↓, caspase-1↓, cleaved caspase-3↓, Bcl-2↑, NLRP3↓, LC3-II/I↓, IL-1β↓, IL-18↓, mTOR↑, Beclin-1↑, p62↑
22	Han et al. (55)	MCAO	GB20	EA (untold)	CACNA1B-CaM-CaMKII-CREB axis and cognitive ability	Neurological deficit score↓, MWM escape latency↓	protein levels of GAP-43, SYN and PSD-95↑, the number of synapses in CA1↑, mRNA levels of CACNA1B, CaM, Ca2+/CaMKII and CREB↑, p-CaM, p-CaMKII and p-CREB↑
23	Su et al. (74)	MCAO/R	GV24, GV20	EA, dense-sparse wave, frequency 2 ~ 10 Hz, intensity 1 mA, 30 min/day, 14 days.	Mitophagy and cognitive ability	Neurologic deficit scores↓, MWM escape latency↓, MWM platform crossing times↑	CA1 neuron number↑, the integrity of mitochondrial ultrastructure↑, Beclin-1↑, Sirt1↑, FOXO3↑, PINK1↑, Parkin↑, LC3 II/I↑, P62↓
24	Jia et al. (9)	BCCAO	GV24, GV20	EA, dense-sparse wave, frequency 2 ~ 15 Hz, intensity 1 ~ 2 mA, 30 min/day, 7 days.	Mitochondrial respiratory dysfunction and cognitive ability	MWM escape latency↓, MWM total distance↓, MWM platform crossing times↑	mitochondrial respiratory complex I, II, III and IV↑, mitochondrial ATP↑, the integrity of mitochondrial ultrastructure↑, AMPKα1↑, FAT/CD36↑, ACC2↓

BCCAO, bilateral common carotid arteries occlusion; pBCCAO, permanent bilateral common carotid arteries occlusion; MA, manual acupuncture; MWM, Morris water maze; ROS, reactive oxygen species; RCR/RCI, respiratory control rate/respiratory control index, =state3/state4; AMPK, adenosine monophosphate-activated protein kinase; FAT, fatty acid translocase; CD36, cluster of differentiation 36; ACC2, acetyl-CoA carboxylase 2; MMP, mitochondrial membrane potential; MID, multi-infarct dementia; CBF, cerebral blood flow; SOD, superoxide dismutase; CuZnSOD, copper and zinc-containing superoxide dismutase; MnSOD, Manganese-containing superoxide dismutase; GSH, reduced glutathione; GSSG, oxidized glutathione; MDA, malondialdehyde; O2-, superoxide anion; MCAO, middle cerebral artery occlusion; EA, electroacupuncture; GSH-PX, glutathione peroxidase; LA, laser acupuncture; IL-6, interleukin-6; 2VO, 2-vessel occlusion; 8-OHdG, 8-hydroxy-2 deoxyguanosine; TXNIP, Thioredoxin interacting protein; NLRP3, nucleotide-binding oligomerization domain, leucine-rich repeat and pyrin domain-containing 3; Glu, glutamate; GluR1, glutamate receptor1; NR, NMDAR, N-methyl-D-aspartic acid receptor; GAP-43, growth-associated protein; SYN, synaptophysin; PSD-95, postsynaptic density protein; CACNA1B, Calcium Voltage-Gated Channel Subunit Alpha1 B; CAM, calmodulin; CaMKII/IV, calmodulin-dependent protein kinase type II/IV; CREB, cyclic adenosine monophosphate response element binding; p-CaM, p-CaMKII and p-CREB, phosphorylation of CaM, CaMKII and CREB; ChAT, choline acetyltransferase; BDNF, brain-derived neurotrophic factor; Bcl-2, B-cell lymphoma 2; Bax, Bcl-2-associated X protein; LTP, long-term potentiation; sEPSC, spontaneous excitatory postsynaptic current; ATP, adenosine triphosphate; NO, nitric oxide; iNOS, inducible nitric oxide synthase; TOMM40, translocase of outer mitochondrial membrane 40; TIMM17A, translocase of inner mitochondrial membrane 17A; Aβ, amyloid β; APP, amyloid precursor protein; COX, cyclooxygenase; AChE, acetylcholinesterase; PI3K, phosphatidylinositol 3-kinase; Akt, protein kinase B; p-Akt, phospho-Akt; mTOR, mammalian target of rapamycin; RUF, relative fluorescence units; AANAT, arylalkylamine N-acetyltransferase; LC3, microtubule-associated protein 1 light chain 3; PINK1, PTEN induced putative kinase 1; Parkin, PARK2, Parkinson’s disease gene 2; ASC, apoptosis-associated speck-like protein; IL-1β, interleukin-1β; IL-18, interleukin-18; Lba-1, lonized calcium-binding adapter molecule 1; PGC-1α, peroxisome proliferator-activated receptor γ coactivator 1 α; NRF-1, nuclear respiration factor 1; TFAM, Mitochondrial transcription factor A; OXPHOS, oxide phosphorylation protein complex; CI-NDUFB8, mitochondrial complex I-NADH dehydrogenase (ubiquinone) 1 beta subcomplex, 8; CIV-MTCO1, cytochrome C oxidase complex IV- mitochondrial cytochrome c oxidase subunit I; CV-ATP5A, mitochondrial ATP synthase Complex V- ATP synthase F1 subunit alpha; MAP2, microtubule associated protein 2; CB1R, cannabinoid receptor 1; Cyto C, Cytochrome C; NOR, new object recognition test; DI, discrimination indices; SIRT1, Sirtuin1; OPA1, optic atrophy 1; DRP1, dynamin-related protein 1; TOMM20, translocase of outer mitochondrial membrane 20 homolog; NOX, nicotinamide adenine dinucleotide phosphate (NADPH) oxidase; 3-NT, 3-nitrotyrosine; AR 1, adenosine receptor 1; FOXO3, Forkhead box O3.

Regarding the effectiveness of different acupuncture modalities and parameters, both MA and EA demonstrate efficacy in ameliorating PSCI by targeting mitochondrial pathways. However, the heterogeneity in stimulation parameters (e.g., needle retention time, electrical frequency, treatment duration) and acupoint combinations makes direct comparisons challenging and complicates the determination of an optimal treatment protocol. For instance, some EA studies applying the same acupoint combination and parameter to different animal models have reported opposing regulatory effects on the related pathways of mitophagy, such as the increase and decrease of PI3K, mTOR, and LC3 II/I (29, 45); while different acupoint combinations and parameters applied to the same animal model also resulted in either the promotion or inhibition of mitophagy, respectively (25, 75). This variability likely contributes to inconsistent results in the magnitude of mitochondrial and cognitive improvements across studies, posing a significant challenge for clinical translation. The lack of standardization hinders the replication of findings and the establishment of clear dose–response relationships.

**Figure 1:**
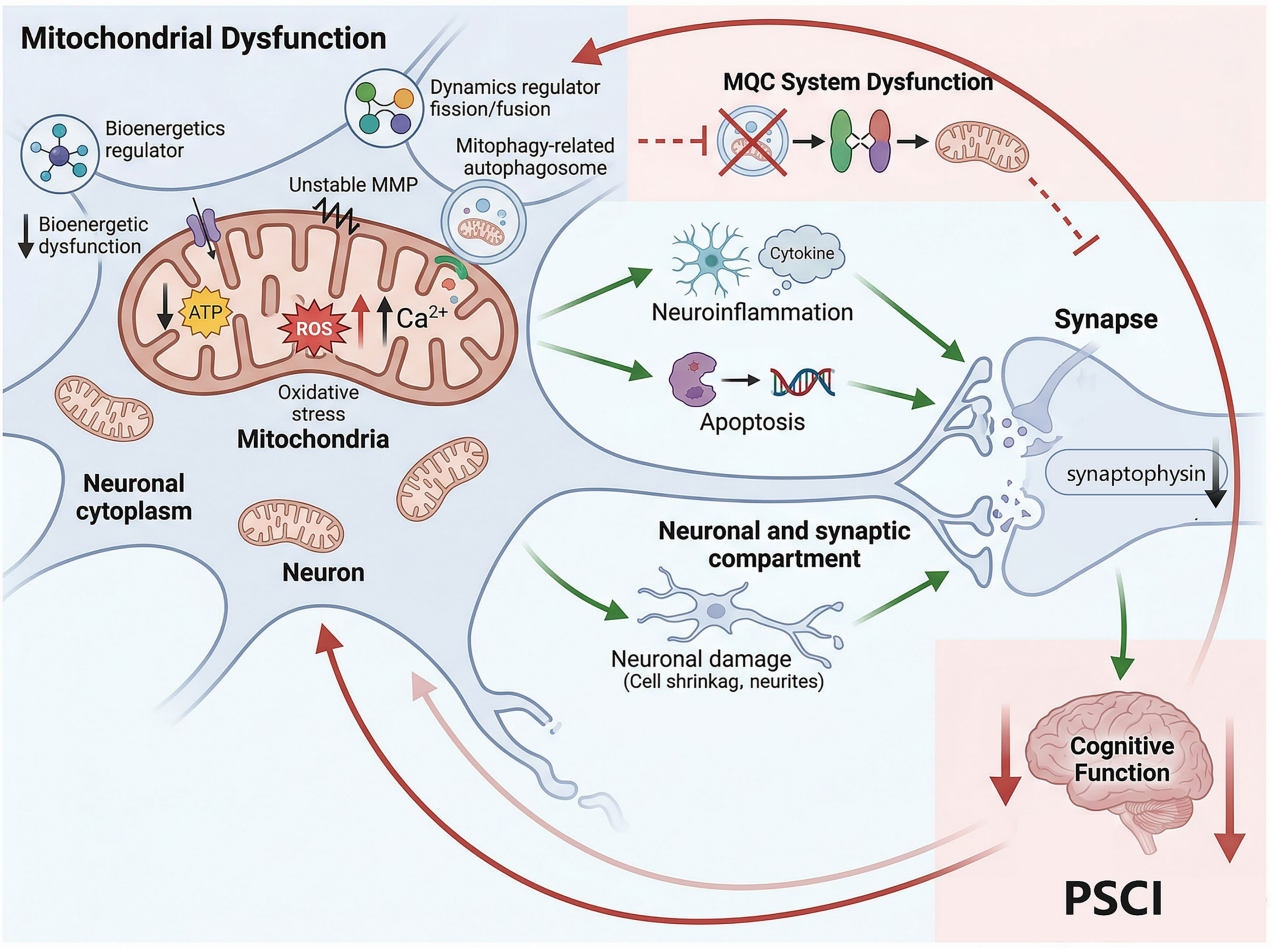
The mitochondrial mechanism of PSCI pathology.

**Figure 2:**
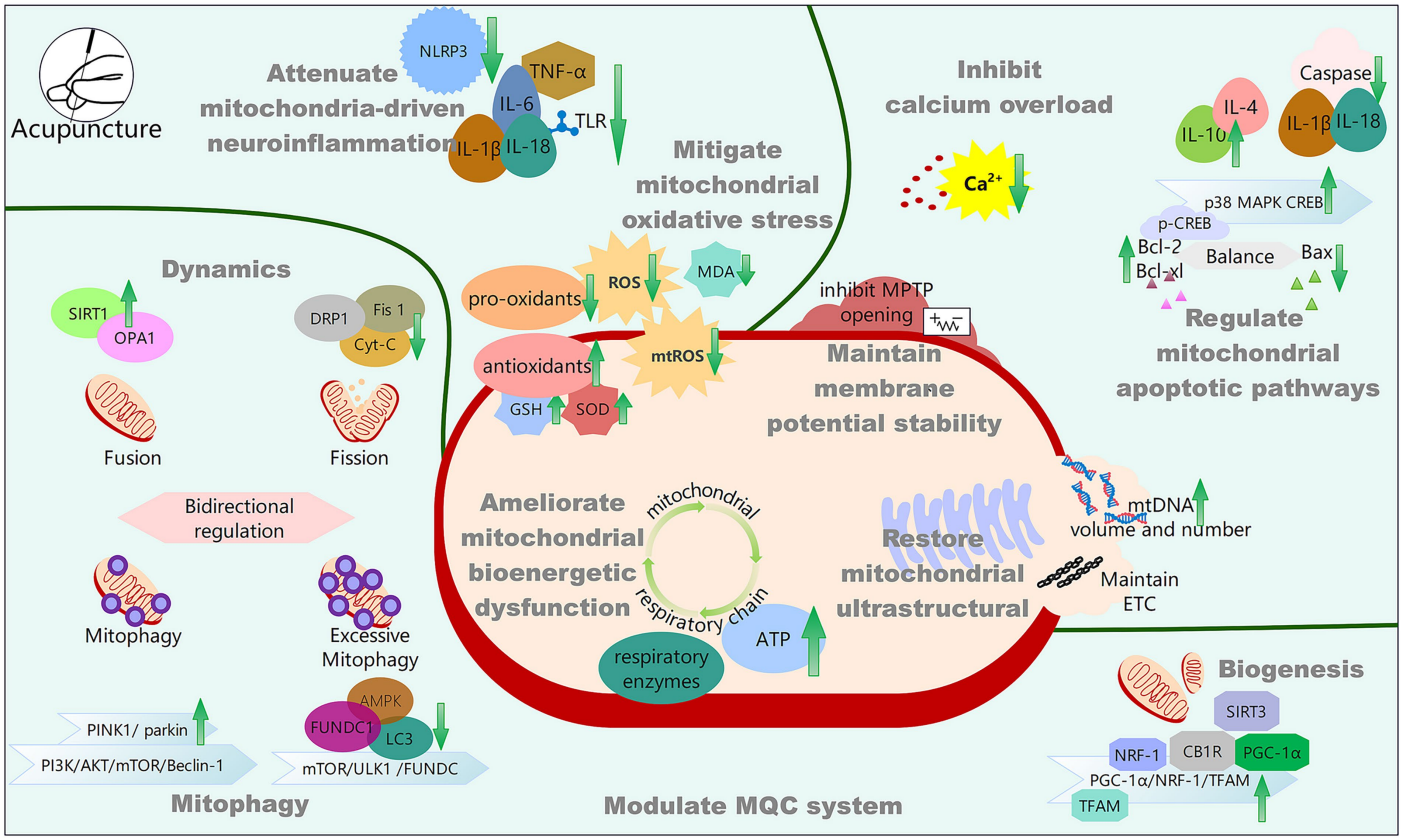
The mitochondrial mechanism of acupuncture effect on PSCI.

Investigating mitochondrial mechanisms offers unique insights for understanding PSCI pathology. Specifically, mitochondrial dysfunction is a core pathological event in PSCI; the mitochondrial pathway represents a more fundamental level of cellular dysfunction and integration (Figure 1). The ability of acupuncture to modulate mitochondrial function provides a perspective for understanding its holistic and systemic therapeutic effects, potentially explaining its concurrent benefits across multiple pathological processes. It offers a unified mechanistic framework that transcends explanations based on single pathways. Furthermore, the clinical guidance and translational value of this review are substantial. It provides a mechanistic rationale for using acupuncture as a complementary therapy for PSCI, highlighting mitochondria as a critical therapeutic target. Acupuncture, as an effective traditional non-pharmaceutical treatment method, contributes to improving PSCI through multi-target regulation of mitochondrial-related functions (Figure 2). Firstly, acupuncture exerts its therapeutic effects by enhancing key mitochondrial functions, including alleviating oxidative stress, correcting energy metabolism disorders, reducing calcium overload, stabilizing MMP, and repairing mitochondrial ultrastructure. Secondly, acupuncture effectively regulates the MQC system, primarily through the bidirectional modulation of mitophagy, with limited evidence currently supporting its role in promoting mitochondrial biogenesis and dynamic balance. Moreover, acupuncture potentially inhibits downstream pathological responses such as neuroinflammation and apoptosis by improving mitochondrial function.

## 6 Limitation and future perspectives

Current evidence indicates that acupuncture may mitigate ischemic injury and cognitive impairment in PSCI by restoring mitochondrial function. Although acupuncture demonstrates multi-target efficacy in improving mitochondrial dysfunction, current evidence is largely confined to improvement in neurological scores and reduction in cerebral infarct volume mediated by single cytokines or mitochondrial pathways post-ischemia, while direct mechanistic links to cognitive function of PSCI remain underexplored. Overall, several limitations of this study must be acknowledged. First, the current evidence is predominantly based on associations. Several previous studies did not employ antagonists to block mitochondrial pathways, which limits the conclusiveness of their findings and renders the direct involvement of mitochondria speculative. There is a pressing need for studies designed to establish direct causation, such as those employing mitochondrial-specific gain- or loss-of-function approaches (e.g., using specific agonists, inhibitors, or genetic models) in conjunction with acupuncture interventions to definitively elucidate causal pathways. Second, the reproducibility of findings is further challenged by heterogeneous animal models, and varying acupuncture parameters, which include differences in stimulation type (e.g., manual vs. EA), frequency, duration, and acupoint combinations. Differences in the therapeutic effects of various acupoint combinations or EA frequencies warrant comparison and analysis; however, the scarcity of existing studies renders such comparisons lacking definitive clinical guidance. Future research should adopt standardized protocols and critical quality evaluations to enhance reproducibility. Common limitations in preclinical models, such as potential performance bias, frequent lack of blinding procedures, and inherent model variability, need to be systematically addressed. Moreover, there is a general scarcity of large-scale, multi-center randomized controlled trials (RCTs) in this field. Therefore, high-quality studies are urgently needed to elucidate the specific mitochondrial mechanisms through which acupuncture improves cognitive outcomes in PSCI. Third, mitochondrial dysfunction occurs across the early, chronic, and progressive stages of cerebral ischemia, with the severity of cognitive decline evolving over time and potentially involving distinct dominant pathogenic factors at different phases. However, the timing of acupuncture interventions remains ambiguous and unstandardized in most current studies. This ambiguity underscores the need to firstly delineate the key stages and pathogenesis of PSCI, which is crucial for determining the optimal intervention window to maximize cognitive recovery.

To guide future research in this field, several key steps are essential. First, conducting comparative studies to systematically evaluate the effects of different acupuncture parameters on specific mitochondrial endpoints is crucial. Second, future research should investigate the temporal dynamics of mitochondrial responses to acupuncture and their correlation with long-term cognitive recovery. Third, the integration of multi-omics approaches may reveal novel pathways related to mitochondrial function. Finally, fostering collaboration between acupuncturists and mitochondrial biologists is pivotal for developing standardized, mechanism-driven treatment protocols. Addressing the current heterogeneity through rigorous standardization is imperative to consolidate knowledge, enhance result consistency, and ultimately accelerate the development of effective, evidence-based acupuncture interventions for PSCI.

## 7 Clinical applicability

While animal studies have established a clear link between acupuncture’s cognitive benefits and mitochondrial mechanisms in vascular dementia models, direct clinical RCT evidence specifically linking mitochondrial mechanisms to acupuncture’s effect in PSCI remains scarce. Existing high-quality RCTs on acupuncture for vascular cognitive impairment have primarily demonstrated the clinical superiority of acupuncture interventions (101, 102). These clinical benefits are supported by mechanistic studies showing acupuncture can regulate peripheral and central inflammation (103), alleviate oxidative stress (104) and inhibit cell apoptosis (105). These processes are closely intertwined with mitochondrial function and have been shown in animals to be downstream effects or related pathways of acupuncture’s regulation of mitochondria. However, in human RCTs, the direct causal chain linking acupuncture intervention to improvement in inflammatory, oxidative stress, or metabolic markers, and subsequently to changes in mitochondrial function, lacks systematic empirical validation. Consequently, direct evidence supporting the mitochondrial mechanism of acupuncture in improving PSCI cognition remains limited.

Translating these promising preclinical findings into clinical practice faces significant hurdles. First, assessing brain mitochondrial function in living patients is profoundly challenging. Preclinical evidence relies on post-mortem brain tissue analysis, a method not feasible in clinical trials. Second, a lack of standardized acupuncture protocols impedes replication and comparison. Studies utilize varied acupoint combinations, stimulation parameters, and treatment durations, complicating the identification of an optimal regimen for mitochondrial modulation. Third, patient heterogeneity introduces complexity far beyond controlled animal models. Factors such as stroke subtype, lesion location, severity, time since stroke, comorbidities, and individual Traditional Chinese Medicine syndrome patterns (106) result in a heterogeneous population in which a uniform mitochondrial response to acupuncture is unlikely, thereby complicating the control of confounding variables and the achievement of consistent results.

To bridge the gap between preclinical mechanisms and clinical application, rigorously designed human trials are essential. Future clinical research must prioritize stringent trial designs, potentially incorporating sham-acupuncture controls and stratified randomization based on biomarkers or syndrome differentiation. Crucially, a biomarker strategy is needed to infer central nervous system mitochondrial status indirectly. This could involve measuring peripheral mitochondrial biomarkers in platelets or extracellular vesicles, an approach aligned with omics technologies used in preclinical work (107). Furthermore, neuroimaging modalities that assess cerebral metabolism and perfusion, such as magnetic resonance spectroscopy or positron emission tomography (108), can serve as *in vivo* proxies for brain energy metabolism. The integration of such objective biomarkers with standardized clinical cognitive assessments will be key to validating the mitochondrial pathway of acupuncture in PSCI patients and guiding personalized, mechanism-informed treatment strategies.

Understanding how acupuncture modulates mitochondria in PSCI could refine its clinical use. This insight could facilitate biomarker-based patient stratification, wherein individuals exhibiting specific mitochondrial dysfunctions may derive greater benefit from acupuncture. However, the reliability of current peripheral biomarkers necessitates further validation. Second, elucidating these mechanisms is essential for optimizing treatment protocols. Preclinical evidence indicates that different EA parameters and acupoint combinations engage distinct pathways; therefore, defining their specific effects on mitochondria could help identify optimal parameters, although rigorous dose–response studies are currently lacking. Third, it supports the rational design of combination therapies. In the context of vascular cognitive impairment, combined acupuncture and pharmacological therapy may outperform monotherapy (102), suggesting potential synergistic effects when paired with mitochondrial-targeting agents or exercise regimens for PSCI (109). Ultimately, while these avenues highlight the path toward a more mechanism-guided strategy, the field still demands high-quality research to substantiate this potential for clinical translation.

## 8 Conclusion

Acupuncture exerts neuroprotective effects in PSCI by regulating mitochondrial function through multi-target and multi-level mechanisms. These mechanisms involve preserving core mitochondrial functions, modulating the MQC system, and potentially influencing downstream pathological processes. Thus, it represents a promising therapeutic strategy for PSCI. Despite initial insights into the relevant mechanisms, current conclusions largely lack direct causal evidence and are constrained by limitations in standardization, temporality, and cognitive correlation. To address these issues, future research necessitates more rigorous causal verification, unified research standards, and cognitively oriented experimental designs. Such efforts will elucidate the precise pathways through which acupuncture regulates mitochondria and ultimately facilitating its clinical translation.

## Glossary

2VO: 2-vessel occlusion
AD: Alzheimer’s disease
Akt: protein kinase B
ATP: adenosine triphosphate
A*β*: amyloid β
Bax: Bcl-2-associated X protein
BCCAO: bilateral common carotid arteries occlusion
Bcl-2: B-cell lymphoma 2
CaM: calmodulin
CaMK: calmodulin dependent protein kinase
CCH: chronic cerebral hypoperfusion
COX: cyclooxygenase
CREB: cyclic adenosine monophosphate response element binding
Cyto C: cytochrome C
DI: discrimination indices
DRP1: dynamin-related protein 1
EA: electroacupuncture
MA: manual acupuncture
MCAO: middle cerebral artery occlusion
MCAO/R: middle cerebral artery occlusion/reperfusion
MDA: malondialdehyde
MID: multi-infarct dementia
MMP: mitochondrial membrane potential
MnSOD: manganese-containing superoxide dismutase
MOMP: mitochondrial outer membrane permeabilization
MPTP: mitochondrial permeability transition pore
MQC: mitochondrial quality control
mtDAMPs: damage-associated molecular patterns
mtDNA: mitochondrial DNA
mtROS: mitochondrial ROS
mTOR: mammalian target of rapamycin
MWM: Morris water maze
NLRP3: nucleotide-binding oligomerization domain, leucine-rich repeat and pyrin domain-containing 3
NOR: new object recognition test
OPA1: optic atrophy 1
OXPHOS: oxide phosphorylation protein complex
pBCCAO: permanent bilateral common carotid arteries occlusion
PD: Parkinson’s disease
PI3K: phosphatidylinositol 3-kinase
PINK1: PTEN induced putative kinase 1
PSCI: Post-stroke cognitive impairment
PRR: pattern recognition receptor
RCD: regulatory cell death
RCT: randomized controlled trial
ROS: reactive oxygen species
SOD: superoxide dismutase
tBCCAO: temporary bilateral common carotid artery occlusion
TFAM: mitochondrial transcription factor A
tMCAO: transient middle cerebral artery occlusion
VD: vascular dementia

## References

[1] El Husseini N Katzan IL Rost NS Blake ML Byun E Pendlebury ST . Cognitive impairment after ischemic and hemorrhagic stroke: a scientific statement from the American Heart Association/American Stroke Association. Stroke. (2023) 54:e272–91. doi: 10.1161/STR.0000000000000430, 37125534 PMC12723706

[2] Stroke Prevention Project NHC Xunming J. Brief report on stroke prevention and treatment in China, 2024. J Capital Med Univ. (2025) 46:947–60. doi: 10.3969/j.issn.1006-7795.2025.06.001

[3] Wang K Dong Q Yu J. Experts consensus on post-stroke cognitive impairment management 2021. Chinese journal of. Stroke. (2021) 16:376–89. doi: 10.3969/j.issn.1673-5765.2021.04.011

[4] Annesley SJ Fisher PR. Mitochondria in health and disease. Cells. (2019) 8:7. doi: 10.3390/cells8070680 PMC6678092 31284394

[5] Tian H Chen X Liao J Yang T Cheng S Mei Z . Mitochondrial quality control in stroke: from the mechanisms to therapeutic potentials. J Cell Mol Med. (2022) 26:1000–12. doi: 10.1111/jcmm.17189, 35040556 PMC8831937

[6] Aliev G Palacios HH Gasimov E Obrenovich ME Morales L Leszek J . Oxidative stress induced mitochondrial failure and vascular Hypoperfusion as a key initiator for the development of Alzheimer disease. Pharmaceuticals. (2010) 3:158–87. doi: 10.3390/ph3010158, 27713247 PMC3991025

[7] Sun L Zhao Z Guo J Qin Y Yu Q Shi X . Mitochondrial transplantation confers protection against the effects of ischemic stroke by repressing microglial pyroptosis and promoting neurogenesis. Neural Regen Res. (2024) 19:1325–35. doi: 10.4103/1673-5374.385313, 37905882 PMC11467935

[8] Yang W Wen W Chen H Zhang H Lu Y Wang P . Zhongfeng Xingnao liquid ameliorates post-stroke cognitive impairment through sirtuin1 (SIRT1)/nuclear factor erythroid 2-related factor 2 (Nrf2)/heme oxygenase 1 (HO-1) pathway. Chin J Nat Med. (2025) 23:77–89. doi: 10.1016/S1875-5364(25)60808-9, 39855833

[9] Jia H Zhang B Han X Yu P Xiong B Liu T . Inhibition of cathepsin B ameliorates murine cognitive dysfunction and neuronal damage in ischemic stroke by inhibiting mitochondrial apoptosis and Drp1-mediated mitochondrial fission. Mol Neurobiol. (2025) 62:12688–704. doi: 10.1007/s12035-025-05094-y, 40447852

[10] Li L Yang L Luo B Deng L Zhong Y Gan D . Acupuncture for post-stroke cognitive impairment: an overview of systematic reviews. Int J Gen Med. (2022) 15:7249–64. doi: 10.2147/IJGM.S376759, 36124104 PMC9482408

[11] Liu Y Chen F Qin P Zhao L Li X Han J . Acupuncture treatment vs. cognitive rehabilitation for post-stroke cognitive impairment: a systematic review and meta-analysis of randomized controlled trials. Front Neurol. (2023) 14:1035125. doi: 10.3389/fneur.2023.1035125, 36846126 PMC9946978

[12] Li N Wang H Liu H Zhu L Lyu Z Qiu J . The effects and mechanisms of acupuncture for post-stroke cognitive impairment: progress and prospects. Front Neurosci. (2023) 17:1211044. doi: 10.3389/fnins.2023.1211044, 37397457 PMC10309044

[13] Li H Liu Y Lin LT Wang XR Du SQ Yan CQ . Acupuncture reversed hippocampal mitochondrial dysfunction in vascular dementia rats. Neurochem Int. (2016) 92:35–42. doi: 10.1016/j.neuint.2015.12.001, 26682902

[14] Jiang YH He JK Li R Chen ZH Jia BH. Mechanisms of acupuncture in improving Alzheimer's disease caused by mitochondrial damage. Chin J Integr Med. (2022) 28:272–80. doi: 10.1007/s11655-022-3511-6 35230607

[15] Ru-qi Z Bing-xu Z Xiao-jie D Lin C Sheng-chun W. Advances of researches on acupuncture treatment of Parkinson's disease by regulating mitochondrial function. Zhen Ci Yan Jiu. (2025) 50:721–7. doi: 10.13702/j.1000-0607.20240406 40551656

[16] Zhao YX Yu XC Gao JH Yao MJ Zhu B. Acupuncture for paclitaxel-induced peripheral neuropathy: a review of clinical and basic studies. J Pain Res. (2021) 14:993–1005. doi: 10.2147/JPR.S296150, 33883931 PMC8055287

[17] Jang JH Song EM Do YH Ahn S Oh JY Hwang TY . Acupuncture alleviates chronic pain and comorbid conditions in a mouse model of neuropathic pain: the involvement of DNA methylation in the prefrontal cortex. Pain. (2021) 162:514–30. doi: 10.1097/j.pain.0000000000002031 32796318 PMC7808350

[18] An H Tao W Liang Y Li P Li M Zhang X . Dengzhanxixin injection ameliorates cognitive impairment through a neuroprotective mechanism based on mitochondrial preservation in patients with acute ischemic stroke. Front Pharmacol. (2021) 12:712436. doi: 10.3389/fphar.2021.712436, 34526899 PMC8435665

[19] Jia XY Li H Rao T You YM Li J Gong YM . Effect of electroacupuncture at “Baihui”(GV20) and “Shenting”(GV24) on cognitive impairment and mitochondrial energy metabolism in vascular dementia rats. Zhen Ci Yan Jiu. (2025) 50:22–31. doi: 10.13702/j.1000-0607.20231097 39961755

[20] Sun C Liu M Liu J Zhang T Zhang L Li H . ShenmaYizhi decoction improves the mitochondrial structure in the brain and ameliorates cognitive impairment in VCI rats via the AMPK/UCP2 signaling pathway. Neuropsychiatr Dis Treat. (2021) 17:1937–51. doi: 10.2147/NDT.S302355, 34168453 PMC8218872

[21] He J Huang Y Du G Wang Z Xiang Y Wang Q. Lasting spatial learning and memory deficits following chronic cerebral hypoperfusion are associated with hippocampal mitochondrial aging in rats. Neuroscience. (2019) 415:215–29. doi: 10.1016/j.neuroscience.2019.04.044, 31055006

[22] Zhao R Xu X Chen P Lu Z Tan G. The changes of mitochondria in hippocampus of vascular dementia rats. Progress in modern. Biomedicine. (2015) 15:2436–9. doi: 10.13241/j.cnki.pmb.2015.13.010

[23] Tang H Bai X Duan C Wu Y Zhang D Ye L . Qufeng Tongqiao Fang influencing vascular dementia rat CA1 pyramidal cell of hippocampus and prefrontal cortex neurons mitochondrial morphological changes. Liaoning. J Tradit Chin Med. (2015) 42:1367. doi: 10.13192/j.issn.1000-1719.2015.06.085

[24] Zhang Y. The Study of Mechanism in Treating the Cognitive Impairment of cerebral Ischemia Reperfusion in rats by Electroacupuncture at Shenting and Baihui [Ph.D. thesis]. Haerbin: University of TCM (2015).

[25] Zhong X Chen B Li Z Lin R Ruan S Wang F . Electroacupuncture ameliorates cognitive impairment through the inhibition of NLRP3 Inflammasome activation by regulating melatonin-mediated Mitophagy in stroke rats. Neurochem Res. (2022) 47:1917–30. doi: 10.1007/s11064-022-03575-3, 35301664

[26] Qiu R Zhang H Deng C Chen D Xu Y Xiong D . Effects of electroacupuncture on ROS-NLRP3 inflammatory pathway and autophagy related proteins in hippocampus of vascular dementia rats. Zhen Ci Yan Jiu. (2022) 47:298–304. doi: 10.13702/j.1000-0607.20210324 35486008

[27] Lou H Yao J Zhang Y Wu X Sun L Wang Y . Potential effect of acupuncture on mitochondrial biogenesis, energy metabolism and oxidation stress in MCAO rat via PGC-1α/NRF1/TFAM pathway. J Stroke Cerebrovasc Dis. (2024) 33:107636. doi: 10.1016/j.jstrokecerebrovasdis.2024.107636 38346661

[28] Chen L Chen S Bai Y Zhang Y Li X Wang Y . Electroacupuncture improves cognitive impairment after ischemic stroke based on regulation of mitochondrial dynamics through SIRT1/PGC-1α pathway. Brain Res. (2024) 1844:149139. doi: 10.1016/j.brainres.2024.149139 39111521

[29] Wang HL Liu FL Li RQ Wan MY Li JY Shi J . Electroacupuncture improves learning and memory functions in a rat cerebral ischemia/reperfusion injury model through PI3K/Akt signaling pathway activation. Neural Regen Res. (2021) 16:1011–6. doi: 10.4103/1673-5374.300454, 33269744 PMC8224106

[30] Wei Y Miao Q Zhang Q Mao S Li M Xu X . Aerobic glycolysis is the predominant means of glucose metabolism in neuronal somata, which protects against oxidative damage. Nat Neurosci. (2023) 26:2081–9. doi: 10.1038/s41593-023-01476-4 37996529

[31] Iwata R Casimir P Erkol E Boubakar L Planque M Gallego López IM . Mitochondria metabolism sets the species-specific tempo of neuronal development. Science. (2023) 379:705. doi: 10.1126/science.abn4705 36705539

[32] Pundik S Xu K Sundararajan S. Reperfusion brain injury: focus on cellular bioenergetics. Neurology. (2012) 79:S44–51. doi: 10.1212/WNL.0b013e3182695a14, 23008411 PMC12443343

[33] Murphy MP. How mitochondria produce reactive oxygen species. Biochem J. (2009) 417:1–13. doi: 10.1042/BJ20081386 19061483 PMC2605959

[34] Zhang X Wu B Nie K Jia Y Yu J. Effects of acupuncture on declined cerebral blood flow, impaired mitochondrial respiratory function and oxidative stress in multi-infarct dementia rats. Neurochem Int. (2014) 65:23–9. doi: 10.1016/j.neuint.2013.12.004, 24361538

[35] Wen P Sun Z Gou F Wang J Fan Q Zhao D . Oxidative stress and mitochondrial impairment: key drivers in neurodegenerative disorders. Ageing Res Rev. (2025) 104:102667. doi: 10.1016/j.arr.2025.102667, 39848408

[36] Martínez-Cué C Rueda N. Cellular senescence in neurodegenerative diseases. Front Cell Neurosci. (2020) 14:16. doi: 10.3389/fncel.2020.00016, 32116562 PMC7026683

[37] Song N Mei S Wang X Hu G Lu M. Focusing on mitochondria in the brain: from biology to therapeutics. Transl Neurodegener. (2024) 13:23. doi: 10.1186/s40035-024-00409-w, 38632601 PMC11022390

[38] Ji J Kline AE Amoscato A Samhan-Arias AK Sparvero LJ Tyurin VA . Lipidomics identifies cardiolipin oxidation as a mitochondrial target for redox therapy of brain injury. Nat Neurosci. (2012) 15:1407–13. doi: 10.1038/nn.3195, 22922784 PMC3697869

[39] Yao Y Wang F Yang X Zang D Yang J Wang Z. Bombesin attenuated ischemia-induced spatial cognitive and synaptic plasticity impairment associated with oxidative damage. Biomed Pharmacother. (2018) 103:87–93. doi: 10.1016/j.biopha.2018.03.155, 29635132

[40] Lin Y Lin R Chen B Yu K. The possible mechanism of electroacupuncture ameliorating learning and memory ability in rats with focal cerebral ischemia/reperfusion via inhibiting oxidative stress. Chinese. J Rehabil Med. (2015) 30:755–60. doi: 10.3969/j.issn.1001-1242.2015.08.001

[41] Jittiwat J. Baihui point laser acupuncture ameliorates cognitive impairment, motor deficit, and neuronal loss partly via antioxidant and anti-inflammatory effects in an animal model of focal ischemic stroke. Evid Based Complement Alternat Med. (2019) 2019:1204709. doi: 10.1155/2019/1204709 30915140 PMC6409074

[42] Du SQ Wang XR Zhu W Ye Y Yang JW Ma SM . Acupuncture inhibits TXNIP-associated oxidative stress and inflammation to attenuate cognitive impairment in vascular dementia rats. CNS Neurosci Ther. (2018) 24:39–46. doi: 10.1111/cns.12773, 29110407 PMC6489958

[43] Su X Wu Z Mai F Fan Z Du S Qian H . 'Governor vessel-unblocking and mind-regulating' acupuncture therapy ameliorates cognitive dysfunction in a rat model of middle cerebral artery occlusion. Int J Mol Med. (2019) 43:221–32. doi: 10.3892/ijmm.2018.3981, 30431067 PMC6257833

[44] Li L Li X Du X. Acupuncture improves cognitive function of vascular dementia rats by regulating PI3K/Akt/mTOR pathway. Zhen Ci Yan Jiu. (2021) 46:851–6. doi: 10.13702/j.1000-0607.200844, 34698459

[45] Luo J Lang J Xu W Wang L Zhao Z Jia J . Electroacupuncture alleviates post-stroke cognitive impairment through inhibiting miR-135a-5p/mTOR/NLRP3 Axis-mediated autophagy. Neuroscience. (2024) 545:185–95. doi: 10.1016/j.neuroscience.2024.03.008, 38522660

[46] Lin R Lin Y Tao J Chen B Yu K Chen J . Electroacupuncture ameliorates learning and memory in rats with cerebral ischemia-reperfusion injury by inhibiting oxidative stress and promoting p-CREB expression in the hippocampus. Mol Med Rep. (2015) 12:6807–14. doi: 10.3892/mmr.2015.4321, 26397995

[47] Ye Y Zhu W Wang XR Yang JW Xiao LY Liu Y . Mechanisms of acupuncture on vascular dementia-a review of animal studies. Neurochem Int. (2017) 107:204–10. doi: 10.1016/j.neuint.2016.12.001, 28034725

[48] Rizzuto R De Stefani D Raffaello A Mammucari C. Mitochondria as sensors and regulators of calcium signalling. Nat Rev Mol Cell Biol. (2012) 13:566–78. doi: 10.1038/nrm3412, 22850819

[49] Jeong SY Seol DW. The role of mitochondria in apoptosis. BMB Rep. (2008) 41:11–22. doi: 10.5483/bmbrep.2008.41.1.011 18304445

[50] Brown GC. Nitric oxide and mitochondrial respiration. Biochim Biophys Acta. (1999) 1411:351–69. 10320668 10.1016/s0005-2728(99)00025-0

[51] Rowland AA Voeltz GK. Endoplasmic reticulum-mitochondria contacts: function of the junction. Nat Rev Mol Cell Biol. (2012) 13:607–25. doi: 10.1038/nrm3440, 22992592 PMC5111635

[52] Paillusson S Stoica R Gomez-Suaga P Lau DHW Mueller S Miller T . There's something wrong with my MAM; the ER-mitochondria Axis and neurodegenerative diseases. Trends Neurosci. (2016) 39:146–57. doi: 10.1016/j.tins.2016.01.008, 26899735 PMC4780428

[53] Kong L Yang J Yang H Xu B Yang T Liu W. Research advances on CaMKs-mediated neurodevelopmental injury. Arch Toxicol. (2024) 98:3933–47. doi: 10.1007/s00204-024-03865-5 39292234

[54] Cohen SM Suutari B He X Wang Y Sanchez S Tirko NN . Calmodulin shuttling mediates cytonuclear signaling to trigger experience-dependent transcription and memory. Nat Commun. (2018) 9:2451. doi: 10.1038/s41467-018-04705-8, 29934532 PMC6015085

[55] Han Q Wang F. Electroacupuncture at GB20 improves cognitive ability and synaptic plasticity via the CaM-CaMKII-CREB signaling pathway following cerebral ischemia-reperfusion injury in rats. Acupunct Med. (2024) 42:23–31. doi: 10.1177/09645284231202805, 38126262

[56] Zhang Y Lin R Tao J Wu Y Chen B Yu K . Electroacupuncture improves cognitive ability following cerebral ischemia reperfusion injury via CaM-CaMKIV-CREB signaling in the rat hippocampus. Exp Ther Med. (2016) 12:777–82. doi: 10.3892/etm.2016.3428, 27446275 PMC4950700

[57] Dai Y Zhang Y Yang M Lin H Liu Y Xu W . Electroacupuncture increases the hippocampal synaptic transmission efficiency and long-term plasticity to improve vascular cognitive impairment. Mediat Inflamm. (2022) 2022:1–15. doi: 10.1155/2022/5985143 PMC9246579 35784174

[58] Kent AC El Baradie KBY Hamrick MW. Targeting the mitochondrial permeability transition pore to prevent age-associated cell damage and neurodegeneration. Oxidative Med Cell Longev. (2021) 2021:6626484. doi: 10.1155/2021/6626484, 33574977 PMC7861926

[59] Qi H Yin YS Yin ZY Li X Shuai JW. Mitochondrial outer membrane permeabilization and inner membrane permeabilization in regulating apoptosis and inflammation. J Theor Biol. (2023) 571:111558. doi: 10.1016/j.jtbi.2023.111558, 37327862

[60] Yapryntseva MA Zhivotovsky B Gogvadze V. Permeabilization of the outer mitochondrial membrane: mechanisms and consequences. Biochim Biophys Acta Mol basis Dis. (2024) 1870:167317. doi: 10.1016/j.bbadis.2024.167317, 38909847

[61] Du H Guo L Zhang W Rydzewska M Yan S. Cyclophilin D deficiency improves mitochondrial function and learning/memory in aging Alzheimer disease mouse model. Neurobiol Aging. (2011) 32:398–406. doi: 10.1016/j.neurobiolaging.2009.03.003, 19362755 PMC3304024

[62] Rao VK Carlson EA Yan SS. Mitochondrial permeability transition pore is a potential drug target for neurodegeneration. Biochim Biophys Acta. (2014) 1842:1267–72. doi: 10.1016/j.bbadis.2013.09.003, 24055979 PMC3991756

[63] Liu BH Xu CZ Liu Y Lu ZL Fu TL Li GR . Mitochondrial quality control in human health and disease. Mil Med Res. (2024) 11:32. doi: 10.1186/s40779-024-00536-5 38812059 PMC11134732

[64] Gureev AP Silachev DN Sadovnikova IS Krutskikh EP Chernyshova EV Volodina DE . The ketogenic diet but not Hydroxycitric acid keeps brain mitochondria quality control and mtDNA integrity under focal stroke. Mol Neurobiol. (2023) 60:4288–303. doi: 10.1007/s12035-023-03325-8 37074549

[65] Yu X Zhang R Wei C Gao Y Yu Y Wang L . MCT2 overexpression promotes recovery of cognitive function by increasing mitochondrial biogenesis in a rat model of stroke. Anim Cells Syst. (2021) 25:93–101. doi: 10.1080/19768354.2021.1915379 PMC8118516 34234890

[66] Pfanner N Warscheid B Wiedemann N. Mitochondrial proteins: from biogenesis to functional networks. Nat Rev Mol Cell Biol. (2019) 20:267–84. doi: 10.1038/s41580-018-0092-0, 30626975 PMC6684368

[67] Chan DC. Mitochondrial dynamics and its involvement in disease. Annu Rev Pathol. (2020) 15:235–59. doi: 10.1146/annurev-pathmechdis-012419-032711 31585519

[68] Cao YL Meng S Chen Y Feng JX Gu DD Yu B . MFN1 structures reveal nucleotide-triggered dimerization critical for mitochondrial fusion. Nature. (2017) 542:372–6. doi: 10.1038/nature21077, 28114303 PMC5319402

[69] Yamaguchi R Lartigue L Perkins G Scott RT Dixit A Kushnareva Y . Opa1-mediated cristae opening is Bax/Bak and BH3 dependent, required for apoptosis, and independent of Bak oligomerization. Mol Cell. (2008) 31:557–69. doi: 10.1016/j.molcel.2008.07.010, 18691924 PMC2636708

[70] Jin JY Wei XX Zhi XL Wang XH Meng D. Drp1-dependent mitochondrial fission in cardiovascular disease. Acta Pharmacol Sin. (2021) 42:655–64. doi: 10.1038/s41401-020-00518-y, 32913266 PMC8115655

[71] He Z Ning N Zhou Q Khoshnam SE Farzaneh M. Mitochondria as a therapeutic target for ischemic stroke. Free Radic Biol Med. (2020) 146:45–58. doi: 10.1016/j.freeradbiomed.2019.11.005 31704373

[72] Ashrafi G Schwarz TL. The pathways of mitophagy for quality control and clearance of mitochondria. Cell Death Differ. (2013) 20:31–42. doi: 10.1038/cdd.2012.81, 22743996 PMC3524633

[73] Xu B Zhu L Chu J Ma Z Fu Q Wei W . Esculetin improves cognitive impairments induced by transient cerebral ischaemia and reperfusion in mice via regulation of mitochondrial fragmentation and mitophagy. Behav Brain Res. (2019) 372:112007. doi: 10.1016/j.bbr.2019.112007, 31238056

[74] Su KQ Lv Z Zhang M Chen LL Gao J. Effects of electroacupuncture on mitochondrial autophagy and Sirt1/FOXO3/PINK1/parkin pathway in rats with learning-memory impairment after cerebral ischemia reperfusion injury. Chinese Acupunct Moxibust. (2025) 45:193–9. doi: 10.13703/j.0255-2930.20231012-k0005 39943761

[75] Lang J Luo J Lang J Wang L Xu W Jia J . Electroacupuncture alleviated post-stroke cognitive impairment via the mTOR/NLRP3-mediated autophagy-inflammatory pathway. Eur J Med Res. (2024) 29:532. doi: 10.1186/s40001-024-02131-9, 39497200 PMC11536957

[76] Marchi S Guilbaud E Tait SWG Yamazaki T Galluzzi L. Mitochondrial control of inflammation. Nat Rev Immunol. (2023) 23:159–73. doi: 10.1038/s41577-022-00760-x, 35879417 PMC9310369

[77] Kroemer G Galassi C Zitvogel L Galluzzi L. Immunogenic cell stress and death. Nat Immunol. (2022) 23:487–500. doi: 10.1038/s41590-022-01132-2 35145297

[78] Huang Y Jiang W Zhou R. DAMP sensing and sterile inflammation: intracellular, intercellular and inter-organ pathways. Nat Rev Immunol. (2024) 24:703–19. doi: 10.1038/s41577-024-01027-3, 38684933

[79] Doblado L Lueck C Rey C Samhan-Arias AK Prieto I Stacchiotti A . Mitophagy in human diseases. Int J Mol Sci. (2021) 22:903. doi: 10.3390/ijms22083903, 33918863 PMC8069949

[80] White MJ McArthur K Metcalf D Lane RM Cambier JC Herold MJ . Apoptotic caspases suppress mtDNA-induced STING-mediated type I IFN production. Cell. (2014) 159:1549–62. doi: 10.1016/j.cell.2014.11.036, 25525874 PMC4520319

[81] Liao Y Cheng J Kong X Li S Li X Zhang M . HDAC3 inhibition ameliorates ischemia/reperfusion-induced brain injury by regulating the microglial cGAS-STING pathway. Theranostics. (2020) 10:9644–62. doi: 10.7150/thno.47651, 32863951 PMC7449914

[82] Otandault A Abraham JD Al Amir Dache Z Khalyfa A Jariel-Encontre I Forné T . Hypoxia differently modulates the release of mitochondrial and nuclear DNA. Br J Cancer. (2020) 122:715–25. doi: 10.1038/s41416-019-0716-y, 31929518 PMC7054557

[83] Sho T Xu J. Role and mechanism of ROS scavengers in alleviating NLRP3-mediated inflammation. Biotechnol Appl Biochem. (2019) 66:4–13. doi: 10.1002/bab.1700, 30315709

[84] Xian H Watari K Sanchez-Lopez E Offenberger J Onyuru J Sampath H . Oxidized DNA fragments exit mitochondria via mPTP- and VDAC-dependent channels to activate NLRP3 inflammasome and interferon signaling. Immunity. (2022) 55:1370–85.e8. doi: 10.1016/j.immuni.2022.06.007, 35835107 PMC9378606

[85] Xu J Núñez G. The NLRP3 inflammasome: activation and regulation. Trends Biochem Sci. (2023) 48:331–44. doi: 10.1016/j.tibs.2022.10.002, 36336552 PMC10023278

[86] Voet S Mc Guire C Hagemeyer N Martens A Schroeder A Wieghofer P . A20 critically controls microglia activation and inhibits inflammasome-dependent neuroinflammation. Nat Commun. (2018) 9:2036. doi: 10.1038/s41467-018-04376-5, 29789522 PMC5964249

[87] Zhou R Yazdi AS Menu P Tschopp J. A role for mitochondria in NLRP3 inflammasome activation. Nature. (2011) 469:221–5. doi: 10.1038/nature09663 21124315

[88] Mi Y Qi G Vitali F Shang Y Raikes AC Wang T . Loss of fatty acid degradation by astrocytic mitochondria triggers neuroinflammation and neurodegeneration. Nat Metab. (2023) 5:445–65. doi: 10.1038/s42255-023-00756-4, 36959514 PMC10202034

[89] Zhao Y Zhang J Zheng Y Zhang Y Zhang XJ Wang H . NAD(+) improves cognitive function and reduces neuroinflammation by ameliorating mitochondrial damage and decreasing ROS production in chronic cerebral hypoperfusion models through Sirt1/PGC-1α pathway. J Neuroinflammation. (2021) 18:207. doi: 10.1186/s12974-021-02250-8, 34530866 PMC8444613

[90] Shimada K Crother TR Karlin J Dagvadorj J Chiba N Chen S . Oxidized mitochondrial DNA activates the NLRP3 inflammasome during apoptosis. Immunity. (2012) 36:401–14. doi: 10.1016/j.immuni.2012.01.009, 22342844 PMC3312986

[91] Zhi H Wang Y Chang S Pan P Ling Z Zhang Z . Acupuncture can regulate the distribution of lymphocyte subsets and the levels of inflammatory cytokines in patients with mild to moderate vascular dementia. Front Aging Neurosci. (2021) 13:747673. doi: 10.3389/fnagi.2021.747673, 34912208 PMC8666891

[92] Xu P Zhang X. Acupuncture for improving neuroinflammation in vascular dementia rats by regulating Th1/Th2 cells balance in peripheral blood. Zhongguo Zhen Jiu. (2022) 42:407–12(in Chinese with English abstract). doi: 10.13703/j.0255-2930.20210330-k0008, 35403400

[93] Jiang T Wu M Zhang Z Yan C Ma Z He S . Electroacupuncture attenuated cerebral ischemic injury and neuroinflammation through α7nAChR-mediated inhibition of NLRP3 inflammasome in stroke rats. Mol Med. (2019) 25:22. doi: 10.1186/s10020-019-0091-4, 31117961 PMC6530013

[94] Green DR. The mitochondrial pathway of apoptosis: part I: MOMP and beyond. Cold Spring Harb Perspect Biol. (2022) 14:38. doi: 10.1101/cshperspect.a041038 PMC9159267 35623793

[95] D'Orsi B Mateyka J Prehn JHM. Control of mitochondrial physiology and cell death by the Bcl-2 family proteins Bax and Bok. Neurochem Int. (2017) 109:162–70. doi: 10.1016/j.neuint.2017.03.010, 28315370

[96] Wang G Tang X Zhao F Qin X Wang F Yang D . Total saponins from Trillium tschonoskii maxim promote neurological recovery in model rats with post-stroke cognitive impairment. Front Pharmacol. (2023) 14:1255560. doi: 10.3389/fphar.2023.1255560, 37745057 PMC10513410

[97] Zhao P Zhang J Kuai J Li L Li X Feng N . TAT-PEP alleviated cognitive impairment by alleviating neuronal mitochondria damage and apoptosis after cerebral ischemic reperfusion injury. Mol Neurobiol. (2023) 60:5655–71. doi: 10.1007/s12035-023-03404-w, 37335462 PMC10471703

[98] Chen B Lin WQ Li ZF Zhong XY Wang J You XF . Electroacupuncture attenuates ischemic brain injury and cellular apoptosis via mitochondrial translocation of Cofilin. Chin J Integr Med. (2021) 27:705–12. doi: 10.1007/s11655-021-3335-4, 33709239

[99] Shi Y Dai Q Ji B Huang L Zhuang X Mo Y . Electroacupuncture Pretreatment prevents cognitive impairment induced by cerebral ischemia-reperfusion via adenosine A1 receptors in rats. Front Aging Neurosci. (2021) 13:680706. doi: 10.3389/fnagi.2021.680706, 34413765 PMC8369428

[100] Yun YC Jang D Yoon SB Kim D Choi DH Kwon OS . Laser acupuncture exerts neuroprotective effects via regulation of Creb, Bdnf, Bcl-2, and Bax gene expressions in the hippocampus. Evid Based Complement Alternat Med. (2017) 2017:7181637. doi: 10.1155/2017/7181637, 28408940 PMC5376935

[101] Wen J Cao Y Chang S Huang Q Zhang Z Wei W . A network meta-analysis on the improvement of cognition in patients with vascular dementia by different acupuncture therapies. Front Neurosci. (2022) 16:1053283. doi: 10.3389/fnins.2022.1053283, 36590305 PMC9797048

[102] Li R Xu C Zhong P Wang K Luo Y Xiao L . Efficacy of acupuncture and pharmacological therapies for vascular cognitive impairment with no dementia: a network meta-analysis. Front Aging Neurosci. (2023) 15:1181160. doi: 10.3389/fnagi.2023.1181160, 37396654 PMC10310406

[103] Zhuan L Yulong C Yamin W Ruidong L Kaiqi S Shuai Y . The clinical mechanism of improvement of cognitive impairment after ischemic stroke through Tongdu Xingshen acupuncture by regulating gut microbes. Modern Trad Chinese Med Mat Med-World Sci Technol. (2025) 27:545–55. doi: 10.11842/wst.20231215006

[104] Xinyi T. Clinical and Mechanism Study on the Treatment of Post-Stroke Cognitive Impairment by Ophthalmic Acupuncture Combined with Tail Moxibustion Based on the Theory of “Activating Governor Vessel for Intelligence Boosting” [Ph.D. Thesis.Liaoning University of Traditional Chinese Medicine (2025). doi: 10.27213/d.cnki.glnzc.2025.000054

[105] Jia-yi L Yan Z Kun W Wei L Guang-xing M Wei S. Clinical effects of Tongfu Xingshen decoction combined with abdominal acupuncture plus warm acupuncture and moxibustion on elderly patients with post-stroke cognitive impairment. Chinese Traditional Patent Medicine. (2024) 46:3997–4001. doi: 10.3969/j.issn.1001-1528.2024.12.015

[106] Ye YS Yang QT Zhu DY Deng KX Lin HJ Zhang X . Effects of moxibustion at Yongquan (KI 1) on cognition function and lower limb motor function in patients with post-stroke cognitive impairment of kidney essence deficiency. Zhongguo Zhen Jiu. (2023) 43:1018–22(in Chinese with English abstract). doi: 10.13703/j.0255-2930.20221104-k0001, 37697876

[107] Chen Y Sun W Sun Z Zhao H Wu T Song Y . Effect of electroacupuncture on hippocampal protein lactylation in a rat model of vascular dementia. Front Neurol. (2025) 16:1629474. doi: 10.3389/fneur.2025.1629474, 40963935 PMC12439496

[108] Yang Y Rao T Jiang Y Zhan Y Cheng J Yin Z . Electroacupuncture ameliorates cognitive impairment and white matter injury in vascular dementia rats via activating HIF-1α/VEGF/VEGFR2 pathway. Neuroscience. (2025) 573:364–80. doi: 10.1016/j.neuroscience.2025.03.063, 40164280

[109] Liu XL Qian ZD Li YX Wang YW Zhang Y Zhang Y . Unveiling synergies: integrating TCM herbal medicine and acupuncture with conventional approaches in stroke management. Neuroscience. (2025) 567:109–22. doi: 10.1016/j.neuroscience.2024.12.043, 39730019

